# Composite Diffraction-Free Beam Formation Based on Iteratively Calculated Primitives

**DOI:** 10.3390/mi14050989

**Published:** 2023-04-30

**Authors:** Pavel A. Khorin, Alexey P. Porfirev, Svetlana N. Khonina

**Affiliations:** 1 Samara National Research University, Samara 443086, Russia; khorin.pa@ssau.ru (P.A.K.); porfirev.alexey@ipsiras.ru (A.P.P.); 2 Image Processing Systems Institute of RAS—Branch of the FSRC “Crystallography and Photonics” RAS, Samara 443001, Russia

**Keywords:** structured laser beams, diffraction optical elements, diffraction-free beam, holographic optical tweezers

## Abstract

To form a diffraction-free beam with a complex structure, we propose to use a set of primitives calculated iteratively for the ring spatial spectrum. We also optimized the complex transmission function of the diffractive optical elements (DOEs), which form some primitive diffraction-free distributions (for example, a square or/and a triangle). The superposition of such DOEs supplemented with deflecting phases (a multi-order optical element) provides to generate a diffraction-free beam with a more complex transverse intensity distribution corresponding to the composition of these primitives. The proposed approach has two advantages. The first is the rapid (for the first few iterations) achievements of an acceptable error in the calculation of an optical element that forms a primitive distribution compared to a complex one. The second advantage is the convenience of reconfiguration. Since a complex distribution is assembled from primitive parts, it can be reconfigured quickly or dynamically by using a spatial light modulator (SLM) by moving and rotating these components. Numerical results were confirmed experimentally.

## 1. Introduction

The term “diffraction-free beam” was introduced to denote a laser beam propagating along the optical axis without changing the transverse distribution, i.e., without the influence of diffraction effects. The most famous among diffraction-free beams are the Bessel modes [1,2,3], which are the solution of the Helmholtz equation in cylindrical coordinates. In addition, Mathieu beams [4,5] are famous for the elliptic coordinate system and parabolic beams [6,7] for the parabolic coordinate system, as well as various generalized beams [8,9,10]. A general property of classical diffraction-free beams is the concentration of the spatial spectrum on a narrow ring. Such a property is often used to generate diffraction-free beams [2,11]. There are also other beams with diffraction-free properties whose spatial spectrum differs significantly from a narrow ring. They include Airy beams, Olver beams, and their modifications [12,13,14,15].

Among the effective methods of forming diffraction-free beams are the applications of axicons [16,17,18], diffraction optical elements (DOEs) [19,20,21], or spatial light modulators (SLMs) [22,23,24]. With these approaches, in contrast to the focusing of a narrow ring [2,11], a significant part of the energy of the incident beam goes to the formation of a diffraction-free beam. However, in some cases, additional coding of the calculated complex amplitudes into a phase-only mask may be required [25,26]. Another simple method of energetically efficient formation of various diffraction-free beams is based on a partial diaphragm of the annular light distribution [27,28], formed, for example, by a tandem of an axicon and a lens [29,30], or a toroidal lens [31,32].

Increased interest in the development and formation of new types of diffraction-free beams is associated with their great utility in various applications, including optical trapping [33,34,35,36], material processing [37,38,39], long-distance self-healing alignment [40,41,42], encryption in optical communications [43,44,45], and increasing depth of field [46,47,48].

Diffraction-free beams play a special role in holographic optical tweezers for controlled laser manipulation of an ensemble of microparticles. Structured laser beams with a complex intensity and phase distribution make it possible to hold and move nano- and micro-sized trapped objects in space. In this case, it is especially important to have the ability not only to form beams with a given transverse field distribution but also to quickly rearrange the structure of the beam. Usually, in this case, SLMs are used for the generation and dynamic movement of a set of focused light points [49,50,51]. Simple optical elements shaping various contour beams [52,53,54] and autofocusing beams [55,56,57,58] are also known. However, this does not ensure the diffraction-free nature of the beams.

The design of optical elements to generate complex diffraction-free patterns, as a rule, requires the use of iterative algorithms [59,60,61,62,63]. Although iterative methods seem to be a universal tool, they do not have global convergence, i.e., only a certain local minimum is guaranteed (one of the possible solutions with some accuracy). The disadvantage of an iterative approach is also the fact of repeated use of the direct and inverse operator at each iteration, which leads to significant time and computational costs.

In this paper, we propose a compromise approach, when a complex image is assembled from a set of diffraction-free primitive pictures (for example, a square or/and a triangle), for which the corresponding DOEs are pre-calculated and optimized using an iterative algorithm based on the narrow ring spatial spectrum. Based on the superposition of such DOEs, taking into account the rotation and displacement of primitives, it is possible to dynamically (for example, using SLM) generate various more complex diffraction-free patterns.

## 2. Methods

### 2.1. Theoretical Background

It is known that a light field bounded by a narrow annular diaphragm is transformed into a diffraction-free beam when focused by a lens. The length of the diffraction-free region in the longitudinal direction is determined by the following parameters [2]:

(1) $$z_{\max}\approx 2R_{0}f/d$$

where *f* is the focal length of the lens, *d* is the middle radius of the annular diaphragm, and the radius *R*_0_ is the smaller value of the lens radius or the effective radius of the diffraction pattern [64]:

(2) $$R_{eff}=\frac{d}{2}+\frac{\lambda f}{\Delta d}$$

where λ is the radiation wavelength, Δ*d* is the width of the ring slit.

Thus, the length of the diffraction-free region defined by Equation (1) decreases both with increasing the radius and the width of the ring slit. Thereby, the diffraction-free beam is formed precisely by a very thin ring (Δ*d →* 0). In an ideal case, when the spatial spectrum is the annular delta function, an arbitrary diffraction-free light field can be described by the Whittaker integral [65,66,67]:

(3) $$E_{ND}(x,y,z)=\exp\left(i\frac{2\pi }{\lambda }\alpha_{z}z\right)\int_{0}^{2\pi }A\;(\varphi )\exp\left[i\frac{2\pi }{\lambda }\alpha_{t}(x\cos\varphi +y\sin\varphi )\right]d\varphi$$

where $\alpha_{t}<1$ is the constant corresponding to the radius of the annular spatial spectrum, $\alpha_{z}=\sqrt{1-\alpha_{t}^{2}}$, and *A*(φ) is an arbitrary angular function on the annular spatial spectrum. Note, the pattern of the angular spectrum may be obtained as the result of an iterative solution to the inverse problem [68].

However, in the case of an infinitely thin spectral ring, energy losses are inevitable, and the convergence of the iterative process of the calculation deteriorates due to the small number of degrees of freedom [59,69,70]. That is why, in the given work, we consider in fact pseudo-diffraction-free beams [61,71], which have all the properties of diffraction-free beams at a certain propagation distance defined by Equation (1) related to the size of the bounding aperture.

### 2.2. Iterative Algorithm

**Figure 1:**
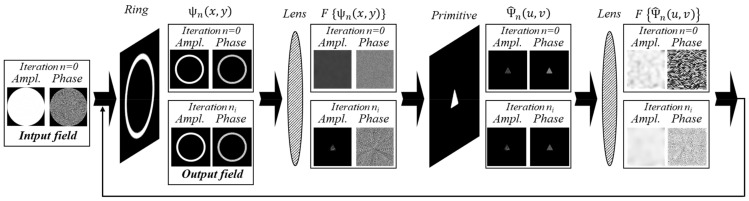
The schematic representation of the iterative algorithm (upper line for the zero iteration and bottom line for *n*th iteration) for the formation of a diffraction-free structured laser beam with a transverse intensity distribution in the form of a given primitive (in particular, “triangle”).

We use an iterative algorithm that was previously successfully used to calculate a DOE shaping a diffraction-free beam of the “light sheet” type [72] suitable for fluorescence microscopy [73,74]. The iterative algorithm is based on the modeling of the action of the lens using the Fourier transform and superimposing the annular diaphragm in the spectrum plane (Figure 1).

**Figure 2:**
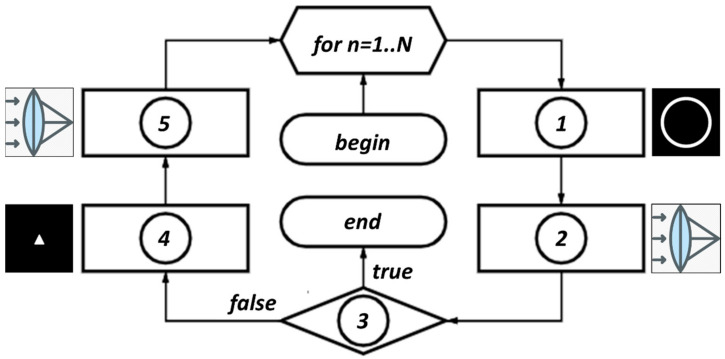
Block diagram of the iterative algorithm, *n* is the iteration number, *N* is the maximum number of iterations, block 1 is the stage with the application of the annular diaphragm, blocks 2 and 5 are the modeling of the lens action using the Fourier transform, block 4 is the stage with the replacement of the intensity according to the shape of the primitive, block 3 is the exit criterion from the iterative algorithm.

The iterative algorithm presented in Figure 1 consists of the following main blocks: truncation of the complex field in the spectral plane by an annular diaphragm, modeling, action of the lens using the Fourier transform and replacement of the intensity by given one in the focal plane. The stopping criterion for the iterative algorithm is determined by the root mean square error (RMSE) of the generated intensity distribution from the given (primitive) picture. When the RMSE falls below the specified threshold *MSE*_0_, the calculation is finished_._ Figure 2 presents a block diagram of an iterative algorithm for the formation of a diffraction-free structured laser beam with a transverse intensity distribution in the form of a given primitive (in particular, “triangle”). The iterative process is described in more detail below.

At the zero iteration *n* = 0, the complex distribution is given in the form of a constant amplitude and a random phase with values in the range from 0 to 2π (“input field” in Figure 1, block “begin” in Figure 2):

(4) $$\psi_{0}(x,y)=\exp[i\;rand(0..2\pi )]$$

Then the input complex field is truncated by an annular diaphragm (${\hat{\psi }}_{n}(x,y)$ in Figure 1, block “1” in Figure 2):

(5) $${\hat{\psi }}_{n}(x,y)=\psi_{n}(x,y)\cdot T(x,y),$$

where *T*(*x,y*) is the ring-function:

(6) $$T(x,y)=\left\{\begin{array}{c} 1,\;\;r_{s}\leq \sqrt{x^{2}+y^{2}}\leq r_{e},\\ 0,\;\;\;else \end{array}\right.$$

where $r_{s}=d-\Delta d/2$, $r_{e}=d+\Delta d/2$.

The annular spectral distribution defined by Equation (5) is Fourier transformed simulating the lens action ($ℑ\{{\hat{\psi }}_{n}(x,y)\}$ in Figure 1, block “2” in Figure 2):

(7) $$\Psi_{n}(u,v)=ℑ\left\{{\hat{\psi }}_{n}(x,y)\right\}=\int_{-\infty }^{\infty }\int_{-\infty }^{\infty }{\hat{\psi }}_{n}(x,y)\exp\left[-2\pi i\left(ux+vy\right)\right]dxdy.$$

The RMSE between the calculated intensity ${\left|\Psi_{n}(u,v)\right|}^{2}$ and the given distribution (primitive) D(u,v) is estimated. If it does not exceed a given value of *MSE*_0_, then the exit criterion (block “3” in Figure 2) is successfully fulfilled, otherwise, we go to the next stage of the algorithm.

At the next stage, the complex distribution $\Psi_{n}(u,v)$ is replaced corresponding to the primitive distribution (“primitive” in Figure 1 and block “4” in Figure 2):

(8) $${\hat{\Psi }}_{n}(u,v)=\frac{\Psi_{n}(u,v)}{\left|\Psi_{n}(u,v)\right|}\cdot \sqrt{D(u,v)}.$$

Next, the Fourier transform is performed again ($ℑ\{{\hat{\Psi }}_{n}(u,v)\}$ in Figure 1, block “5” in Figure 2):

(9) $${\tilde{\psi }}_{n}(x,y)=ℑ\{{\hat{\Psi }}_{n}(u,v)\}.$$

The jump to the next iteration is carried out either at this step:

(10) $$\psi_{n+1}(x,y)={\tilde{\psi }}_{n}(x,y),$$

or after reducing the input field to a pure phase distribution:

(11) $$\psi_{n+1}(x,y)=\exp\left(i\arg\left[{\tilde{\psi }}_{n}(x,y)\right]\right)$$

In the second case, the convergence will be worse due to the loss of part of the degrees of freedom. Therefore, we used the first option in accordance with expression (10). However, in this case, at experimental implementation, additional encoding of amplitude information into phase information will be required [24,25,26,75].

Note that the proposed algorithm is very simple and will work effectively for fairly elementary distributions D(u,v), i.e., precisely for “primitives”. We propose to form a more complex image from a set of various primitive pictures (taking into account rotation and displacement), for which the corresponding generating DOEs have already been calculated. This approach is convenient when using SLMs for the dynamic reconstruction of diffraction-free pictures, for example, in optical trapping [33,34,35,36] and laser material processing [37,38,39].

## 3. Calculation Results

### 3.1. Simple Primitives

**Figure 3:**
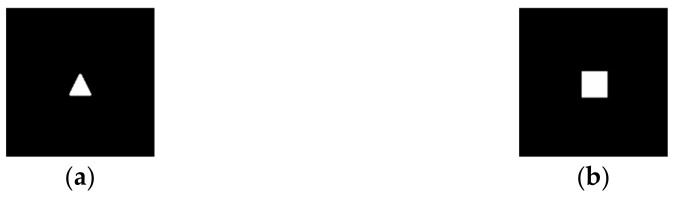
Considered primitives: (**a**) “triangle”, (**b**) “square”.

This section presents the results of calculating the complex transmission function ψ(x,y) using the iterative algorithm described in Section 2.1 with the aim of forming various primitive diffraction-free distributions *D*(*u,v*), for example, a triangle and a square (Figure 3).

Table 1 shows the transverse distribution of the intensity ${\left|\Psi_{n}(u,v)\right|}^{2}$ of the calculated field with a “triangle”-type primitive for different numbers of iterations *n.* It can be seen that the iterative algorithm after some number of iterations comes to a stagnation state, i.e., further iterations do not lead to significant changes.

**Figure 4:**
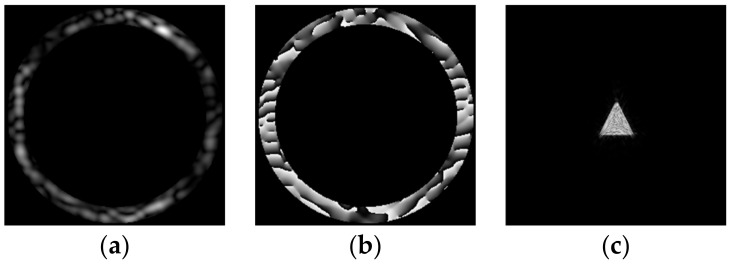
Amplitude (**a**) and phase (**b**) for $\psi_{n=90}(x,y)$ generating a diffraction-free beam (**c**) with a given transverse intensity distribution in the form of a triangle.

The complex transmission function $\psi_{n}(x,y)$ and the corresponding intensity distribution ${\left|\Psi_{n}(u,v)\right|}^{2}$ at the *n* = 90 iteration are presented in Figure 4.

**Figure 5:**
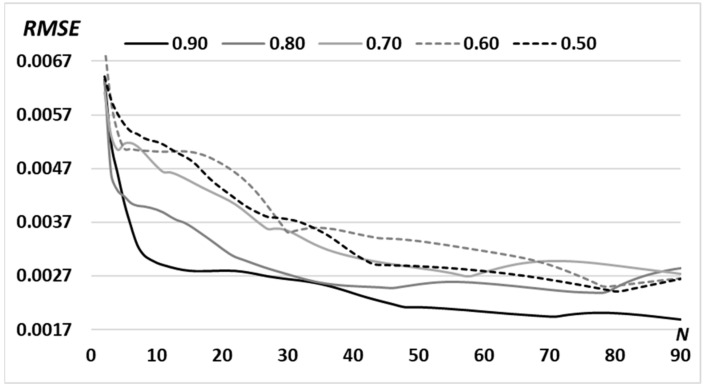
RMSE for the formed intensity distribution from the given (“triangle”-type primitive) for the *N* = 90 iterations with a variable middle radius of the ring *d:* 0.90 *R*_0_; 0.80 *R*_0_; 0.70 *R*_0_; 0.60 *R*_0_; 0.50 *R*_0_ (*R*_0_ is defined in Section 2.1).

To optimize the characteristics of the formed field, variations of the average radius of the circular spectrum *d* were considered (Table 2) at a fixed width Δ*d.* The calculation results for the “triangle” primitive (Figure 5) show that increasing the radius of the ring *d* allows improving the convergence of the algorithm and minimizing the achieved RMSE for a given number of iterations. This is obviously due to an increase in the number of points inside the ring, which leads to an increase in the number of degrees of freedom. Note, the large radius of the spectral ring allows us to take into account high frequencies and clearly describe small details, for example, the borders of the primitive.

Obviously, the larger the slit width (area), the higher the energy efficiency of the beam, and more pixels (degrees of freedom) can be used to improve the convergence of the algorithm. As can be seen from the numerical calculations (Table 2), an increase in the width of the ring slit Δ*d* leads to a decrease in RMSE. However, the diffraction-free properties of the beam will deteriorate. This clearly follows from Equations (1) and (2), since the length of the diffraction-free region *z*_max_ decreases with the increase of the ring slit width Δ*d*. Recall that an ideal diffraction-free beam is formed at an infinitely narrow ring (Δ*d →* 0) [65,66,67].

Therefore, in order to preserve the diffraction-free properties, we chose another way to increase the area of the ring, namely by increasing the middle ring radius *d*, keeping Δ*d* with a small fixed size. As the results of the calculations show (Table 3), increasing the middle ring radius *d* with a fixed ring slit width Δ*d* also leads to a decrease in RMSE, although not so significantly. This is an inevitable compromise between providing diffraction-free properties and forming a given image with acceptable accuracy.

**Figure 6:**
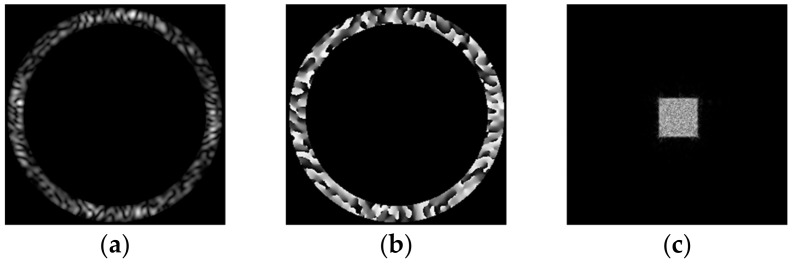

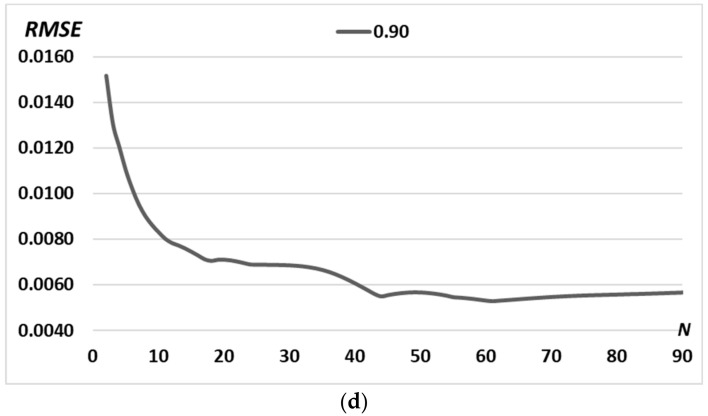
Calculations for the “square”-type primitive *D*(*u, v)*: amplitude (**a**) and phase (**b**) of the generating field $\psi_{n=90}(x,y)$ and the corresponding transverse intensity distribution in the focal plane ${\left|\Psi_{n=90}(u,v)\right|}^{2}$ (**c**). Graph of RMSE versus the number of iterations (**d**).

In a similar way, we calculated the complex transmission function $\psi_{n=90}(x,y)$ for the “square”-type primitive *D*(*u,v*) (Figure 6). From the comparison of the RMSE graph for a “square” (Figure 6d) and a “triangle” (Figure 5), it can be seen that error increases with the increase in the area of the primitive.

**Figure 7:**
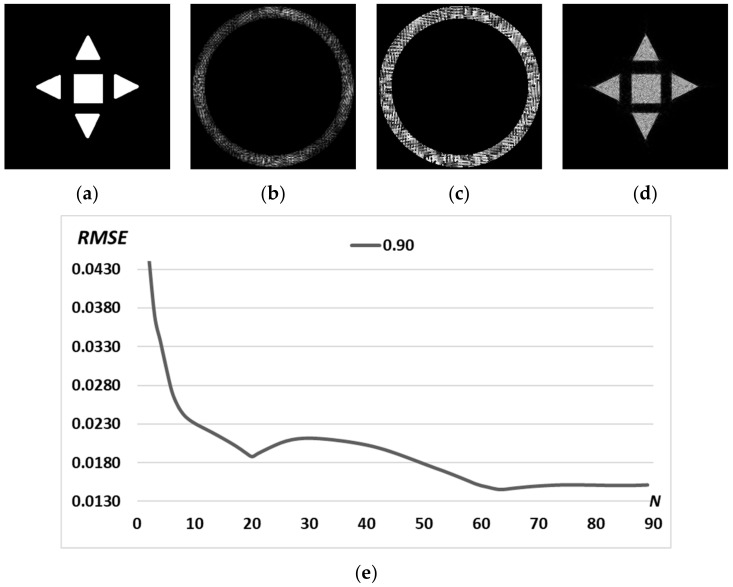
Picture “sign” (**a**); amplitude (**b**) and phase (**c**) of the generating field $\psi_{n=90}(x,y)$ and the corresponding transverse intensity distribution in the focal plane ${\left|\Psi_{n=90}(u,v)\right|}^{2}$ (**d**). Graph of RMSE versus the number of iterations (**e**).

**Figure 8:**
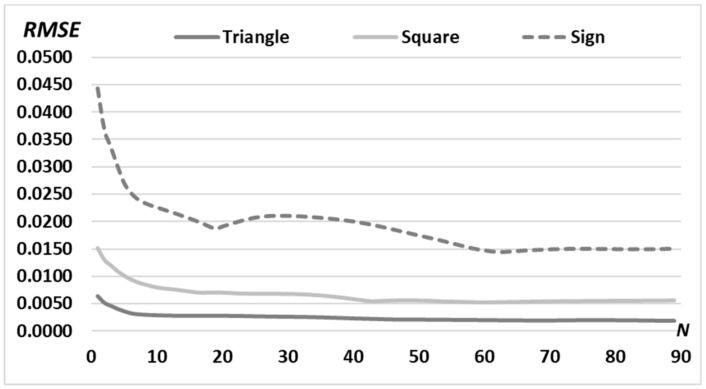
RMSE of the generated intensity distribution for “triangle” (dark solid line) and “square” (light solid line) primitives and for complex “sign” picture (dashed line) for *N* = 90 iterations with a fixed middle radius of the ring *d* = 0.90 *R*_0_.

Next, we target to form complex diffraction-free beams based on the superposition of already calculated primitives. To show the advantage of this approach, let us first consider the iterative calculation of a complex picture, for example, the “sign” picture (Figure 7a), consisting of four triangles and one square. The calculation results are shown in Figure 7. Obviously, the RMSE for complex pictures is greater than the RMSE for individual primitives (Figure 5 and Figure 6d). A more detailed comparison is shown in Figure 8.

### 3.2. Complex Patterns

**Figure 9:**
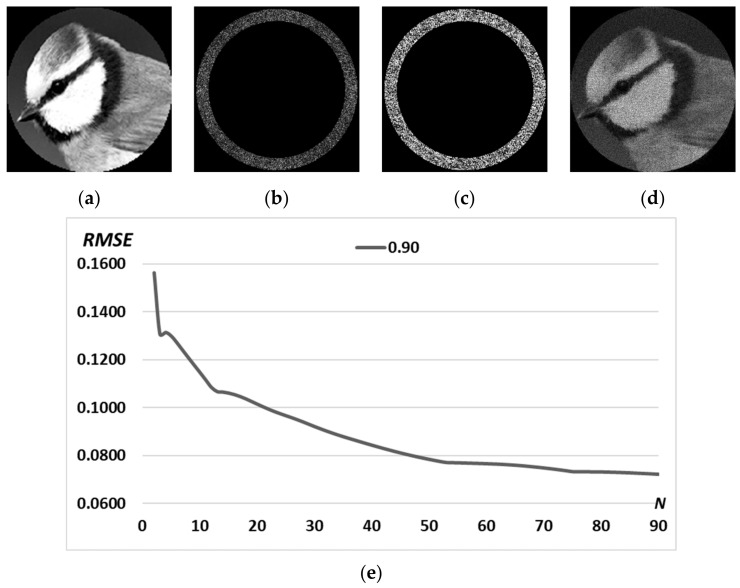
Complex pattern *D*(*u,v*) according to the grayscale image “bird” (**a**); amplitude (**b**) and phase (**c**) of the calculated DOE $\psi_{n=90}(x,y)$ and corresponding transverse intensity distribution in the focal plane ${\left|\Psi_{n=90}(u,v)\right|}^{2}$ (**d**). Graph of RMSE versus the number of iterations (**e**).

For the formation of a diffraction-free beam with a complex predetermined transverse intensity distribution in the form of a grayscale image, we use the same iterative algorithm. Figure 9 presents a complex pattern *D*(*u,v*) with a grayscale “bird” image; amplitude and phase of the calculated DOE $\psi_{n=90}(x,y)$, and the corresponding transverse intensity distribution in the focal plane ${\left|\Psi_{n=90}(u,v)\right|}^{2}$.

**Figure 10:**
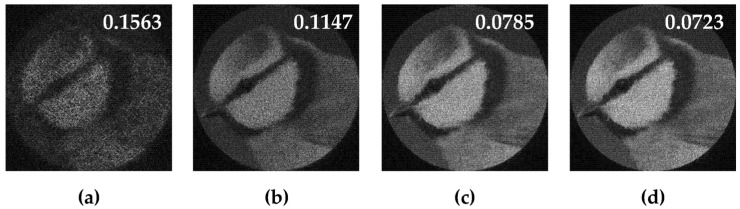
Transverse intensity distribution of the diffraction-free beam ${\left|\Psi_{n}(u,v)\right|}^{2}$ for the complex pattern *D*(*u,v*) of the “bird” image for different numbers of iterations *n: n* = 2 (**a**), *n* = 10 (**b**), *n* = 50 (**c**), *n* = 90 (**d**).

The results of the calculation of a DOE shaping the light field with the complex pattern “bird” (Figure 9a) with different numbers of iterations are shown in Figure 10. The resulting intensity distribution ${\left|\Psi_{n=10}(u,v)\right|}^{2}$ at *n* = 10 iteration has RMSE equal to 0.1147, which is insufficient for the exact reproducibility of the structured beam. The algorithm comes to stagnation approximately at the *n* = 50 iteration with RMSE = 0.0785, so at *n* = 90, the RMSE decreased slightly to 0.0723.

Thus, for complex patterns, RMSE minimization requires significantly more iterations with RMSE values remaining significant (Figure 9) compared to RMSE for primitive patterns (Figure 8).

### 3.3. Superposition of Simple Primitives

In this section, we consider the construction of a diffraction-free beam with a complex predetermined transverse intensity distribution based on the superposition of previously calculated primitives. This method is effective for the optical trapping and manipulation of particles [33,34,35,36], as it provides dynamic restructuring of the configuration of optical traps [35,49,50,51].

To design a multi-order DOE matched with several primitives (such as in Section 3.1), we use the method of spatial carrier frequencies [76]. It is known from the theory of diffraction and the properties of the Fourier transform that if the complex transmission function of DOE ψ(x,y) is multiplied by the carrier function $\exp\left[i\left(ax+by\right)\right]$, then in the focal plane of the element the intensity distribution:

(12) $${\left|\Psi (u,v)\right|}^{2}={\left|ℑ\left(\psi (x,y)\exp\left[i\left(ax+by\right)\right]\right)\right|}^{2}$$

will be shifted by a distance proportional to *a* along the *x*-axis and by a distance proportional to *b* along the *y*-axis. The rotation of the complex function ψ(x,y) by φ_0_ degrees corresponds to the rotation of the intensity distribution ${\left|\Psi (u,v)\right|}^{2}$ by φ_0_ degrees also.

We consider the multi-order DOE as a superposition of DOEs calculated for different primitives $\psi^{\left(p\right)}(x,y)$ taking into account both the rotation angles φ_0p_ and the displacements (*a*_p_, *b*_p_):

(13) $$\tau (x,y)=\sum_{p=0}^{P}\psi^{\left(p\right)}(x,y)\exp\left[i\left(a_{p}x+b_{p}y\right)\right].$$

**Figure 11:**
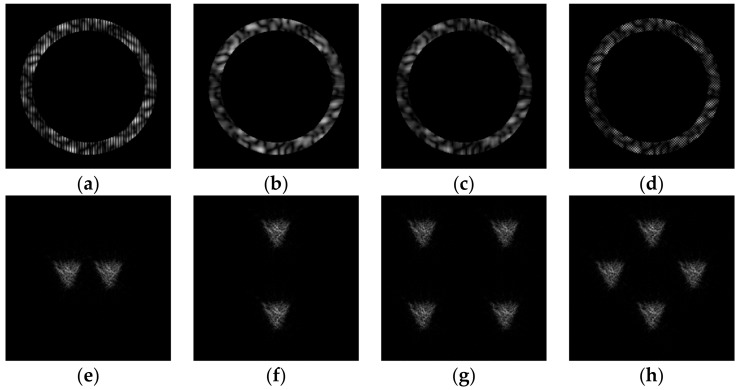
Action of the multi-channel DOEs τ(x,y) calculated from Equation (13) and matched with the “triangle” primitives: amplitude of DOE (**a**–**d**) and corresponding transverse intensity distributions ${\left|ℑ\left\{\tau (u,v)\right\}\right|}^{2}$ (**e**–**h**).

Figure 11 shows the results of the action of the multi-channel DOEs τ(x,y) calculated by Equation (12) and matched with the “triangle” primitives.

**Figure 12:**
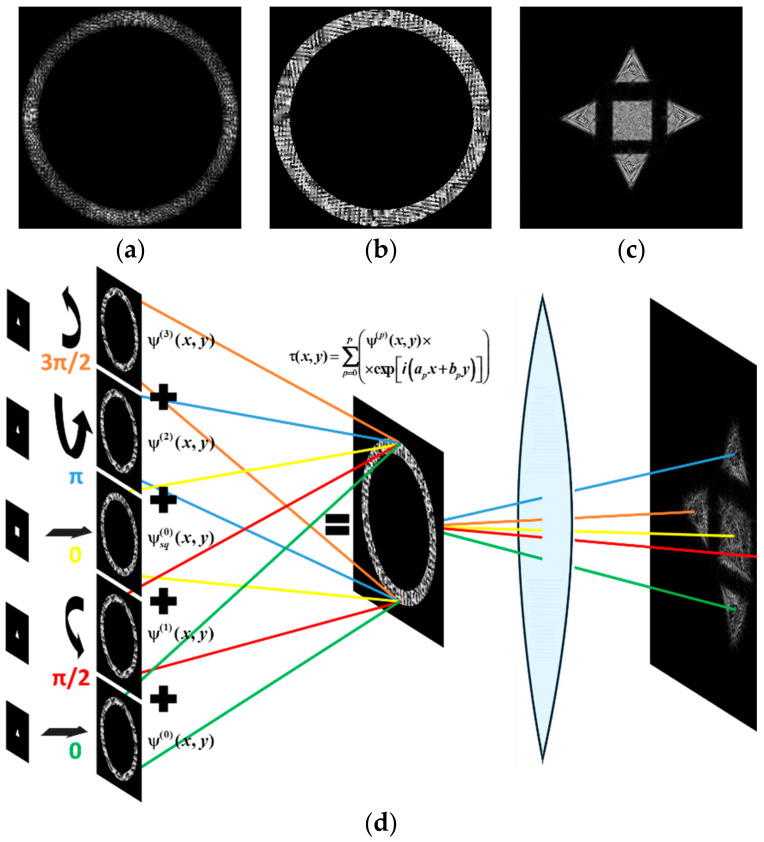
The amplitude (**a**), phase (**b**) of the composed multi-channel DOE, the corresponding transverse intensity distribution shaped in the focal plane (**c**), and the synthesis scheme (**d**).

Obviously, we can compose the multi-channel DOE τ(x,y) calculated by Equation (12) from different types of primitives. Figure 12 shows the multi-channel DOE matched with one “square”-type primitive at the central part and four a “triangle”-type primitives rotated by angles φ_0p_ = πp/2 and shifted from the central part. Figure 12d presents the synthesis scheme for such multi-channel DOE in detail.

## 4. Simulation and Experimental Results

### 4.1. Diffraction-Free Properties

To visualize the diffraction-free properties of a beam generated by the multi-channel DOE composed of the superposition of DOEs calculated for different primitives (Equation (13)), we will calculate the transverse intensity distribution at different distances from the focal plane of the lens.

Such intensity distribution can be calculated using the Fourier transform with the addition of the defocusing function as follows:

(14) $$\begin{array}{l} \Psi_{z}(u,v)=ℑ\left\{\psi (x,y)\exp\left[i\frac{\pi }{\lambda z}(x^{2}+y^{2})\right]\right\}=\\ =\int_{-\infty }^{\infty }\int_{-\infty }^{\infty }\psi (x,y)\exp\left[i\frac{\pi }{\lambda z}(x^{2}+y^{2})\right]\exp\left[-i\frac{2\pi }{\lambda f}\left(ux+vy\right)\right]dxdy, \end{array}$$

where *f* is the focal length and *z* is the defocusing distance.

Table 4 presents a comparison of numerical modeling results for a primitive “triangle” and for the composed diffraction-free beam (superposition of simple primitives) defocused from the focal plane (*f* = 100 mm) on different distances.

It can be seen from Table 4 that a diffraction-free beam based on the “triangle” primitive with a slit width Δ*d* = 0.2 *R*_0_ (1st line) retains its structure better than for Δ*d* = 0.4 *R*_0_ (2nd line): the deviation between the intensity distributions in the focal plane (Δ*z* = 0) and at a distance Δ*z* = −75 mm to the focus is RMSE*z* = 0.0072 (for Δ*d* = 0.2 *R*_0_) and RMSE*z* = 0.009 (for Δ*d* = 0.40 *R*_0_).

Table 4 shows that the composed diffraction-free beam (3^rd^ line) keeps well enough its structure up to 75 mm from the focal plane: the deviation between the intensity distributions in the focal plane (Δ*z* = 0) and at a distance Δ*z* = −75 mm before the focus does not exceed 0.07.

**Figure 13:**
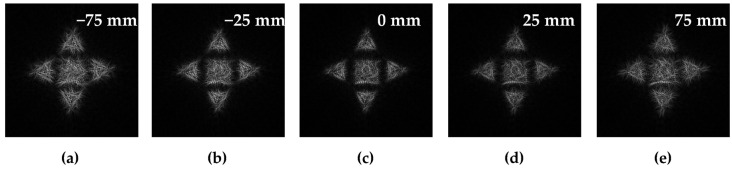
Cross-sectional intensity distribution of a diffraction-free beam given by the superposition of a triangle-type primitive with a different angle of rotation and a square-type primitive at a distance of −75 mm (**a**), −25 mm (**b**), 0 mm (**c**), 25 mm (**d**), and 75 mm (**e**) from the focal plane of a lens with a focus of 100 mm.

We expect that the generated diffraction-free beam retains its structure also after the focal plane. The RMSE between the intensity distribution at the focal plane and the intensity distribution defocused at 75 mm distance (after focus) does not exceed 0.06. The calculation results for the generated diffraction-free beam at defocus distance *z* in the range from −75 mm to 75 mm are presented in Figure 13.

Table 5 presents a comparison of numerical modeling results for a complex pattern of the “bird” image defocused from the focal plane (*f* = 100 mm) on different distances. It can be seen that a diffraction-free beam based on the complex pattern of the “bird” with a slit width Δ*d* = 0.2 *R*_0_ (1st line) in the focal plane has a larger deviation from the given pattern *D*(*u,v*), than for Δ*d* = 0.4 *R*_0_ (2nd line): RMSE = 0.07 and RMSE = 0.05, respectively. However, in the first case, when defocusing, the structure is better preserved: the deviation between the intensity distributions in the focal plane and at a distance Δ*z* = −50 mm to the focus is RMSE*z* = 0.16 and RMSE*z* = 0.19, respectively.

### 4.2. Experimental Results

**Figure 14:**
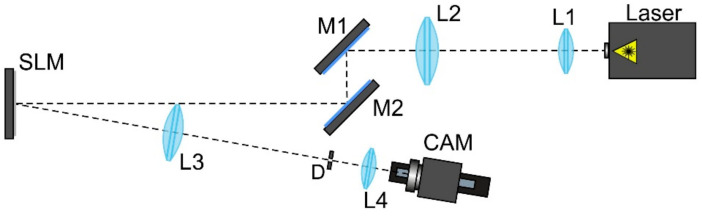
The optical scheme for investigation of the designed DOEs forming diffraction-free laser beams: Laser is a solid-state laser (λ = 532 nm); L1, L2, L3, and L4 are spherical lenses (*f*_1_ =25 mm, *f*_2_ =150 mm, *f*_3_ =500 mm, *f*_4_ =125 mm); M1 and M2 are mirrors, SLM is a reflective spatial light modulator (HOLOEYE PLUTO VIS); D is a circular diaphragm, CAM is a video camera (TOUPCAM UHCCD00800KPA, 3264 × 2448 pixels, 1.67 μm pixel size) mounted on an optical rail.

For the experimental investigation of the designed DOEs, the optical scheme based on a reflective spatial light modulator (SLM) HOLOEYE PLUTO VIS (1920×1080 pixels, pixel size of 8 μm) was used (see Figure 14).

A Gaussian beam from a solid-state laser (λ = 532 nm, *P_out_* = 20 mW) was extended and collimated with a combination of two lenses L1 and L2 with focal lengths of 25 and 150 mm. The collimated laser beam was directed onto the SLM with the help of mirrors M1 and M2. The optical system consisting of two lenses L3 and L4 with focal lengths of 500 and 125 mm as well as a circular diaphragm D was used for spatial filtering of the modulated laser beam reflected from the SLM. Then a video camera CAM (TOUPCAM UHCCD00800KPA; 1600 × 1200 pixels, with a pixel size of 3.34 μm) mounted on an optical rail was used to record the intensity distributions of the formed laser beam at different distances. In the experiments, we used a pure-phase DOE resulting from the encoding of the amplitude-phase transmission function presented in Figure 12.

For the encoding, we used the partial encoding method [75]. Similar to other techniques of amplitude encoding, the used method is sensitive to the inhomogeneity and irregularity of an encoded amplitude distribution. Of course, this introduces the encoding error. In fact, in our work, we used the amplitude encoding only to encode zero amplitude values in the central region of the elements (which have a localized ring structure) to direct light from this region to higher diffraction orders. A limitation of this encoding method is the pixel resolution of the SLM or the manufacturing resolution of the fabricated DOE used to implement the encoded phase mask because the number of pixels increases during coding.

**Figure 15:**
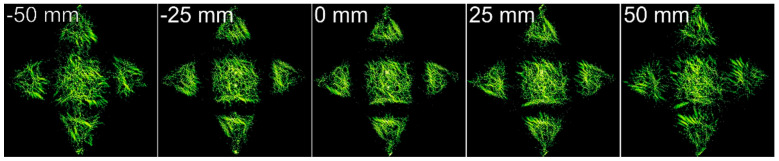
The intensity distributions experimentally generated using the encoded DOE at different distances. The image size is 5 by 5 mm.

The intensity distributions formed by the investigated pure-phase DOE are shown in Figure 15. The experimentally obtained results are in good agreement with numerically obtained ones.

The experimental results qualitatively confirmed numerical calculations and demonstrated the possibility of implementing the calculated ring distribution (when the informative part of the field is concentrated in a narrow region) using SLM, a device with a fixed resolution and number of pixels.

## 5. Discussion

**Figure 16:**
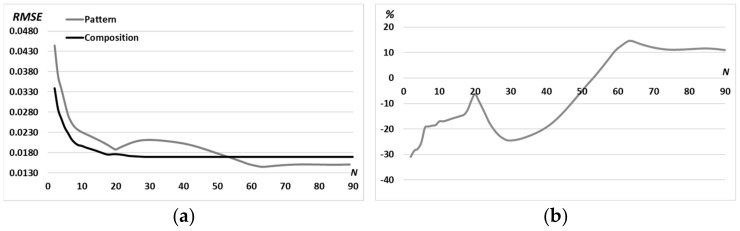
Comparison of the RMSE for the “sign”-pattern (referred to as “pattern”) and the superposition of primitives composed as “sign”-picture (referred to as “composition”) (**a**) and difference in percentage (**b**) for *N* = 90 iterations with a fixed middle ring radius *d* = 0.9*R*_0_.

Let us compare the RMSE for the “sign”-pattern (referred to as “pattern”) and the superposition of primitives composed as “sign”-picture (referred to as “composition”) when we used the iterative algorithm with *N* = 90 iterations (Figure 16).

As seen from Figure 16, the use of the multi-order DOE of Equation (13) composed of primitives in comparison with a DOE calculated for the complex pattern makes it possible to reduce the RMSE already at the first iteration by almost 30% (from 0.044 to 0.034). Moreover, the advantage in RMSE remains during 50 iterations. After the 50^th^ iteration, the situation changes since the iterative algorithm for primitives enters stagnation (after about the 30^th^ iteration, Figure 16a). Thus, the use of a composition of primitives is convenient due to the rapid (for the first few iterations) achievements of an acceptable error (less than 0.02). Changing the structure of the composition does not require additional iterative calculation, as it is achieved by simple operations of rotation and displacement of individual parts of the composition.

From a practical point of view, the results obtained in the article will make it possible to carry out optimization structured light traps, introducing dynamic corrections to the formed intensity distribution with diffraction-free properties.

## 6. Conclusions

In this work, an iterative algorithm based on a ring spectrum and a lens was developed for the formation of a complex structure diffraction-free beam. DOEs were calculated and studied for various values of the parameters responsible for the number of iterations and the radius of the circular spectrum. It was shown that, for primitive patterns, the iterative algorithm provides the rapid (for the first few iterations) achievement of an acceptable RMSE (<0.02) and then comes to a stagnation state when further iterations do not lead to significant changes in the error. The calculated RMSE does not exceed 0.006 for a triangle-type primitive, 0.015 for a square-type primitive, 0.06 for a superposition of primitives, and 0.07 for a complex grayscale image.

To calculate the structured laser beam given from the superposition of primitives, a multi-order DOE based on the method of spatial carrier frequencies was developed. A diffraction-free beam with a more complex transverse intensity distribution was formed based on the superposition of the DOEs pre-calculated for different primitives (taking into account rotation and shifting). The proposed method of synthesis of multi-order DOEs is convenient due to the rapid or dynamic (when using SLM) reconfiguration of the composition structure without additional iterative calculation, as it is achieved by simple operations of rotation and displacement of individual parts of the composition.

To visualize the diffraction-free properties of the calculated beam, the transverse distribution of the intensity at different distances from the focal plane of the lens was calculated. We estimated that the RMSE between the focal intensity distribution (for a lens with a focal length of 100 mm) and the intensity at a distance of 75 mm from the focal plane does not exceed 0.07. To test the diffraction-free properties of the calculated beam, an optical experiment was carried out and the intensity distributions were recorded before and after passing through the lens. The experimentally obtained results are in good agreement with the numerical ones.

## Figures and Tables

**Table 1:** Simulation results for iterative algorithm with primitive “triangle”.

Iteration, *n*	2	4	10	50	90
Intensity	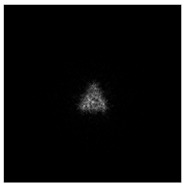	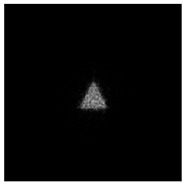	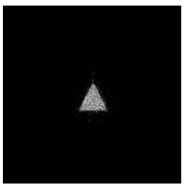	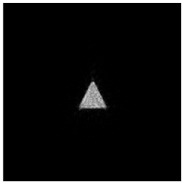	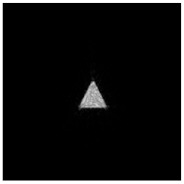
RMSE	0.0064	0.0046	0.0029	0.0021	0.0019

**Table 2:** DOE phase $\psi_{n=90}(x,y)$ with fixed maximal outer ring radius $R_{0}$ and variable width of the ring slit Δ*d* to form a diffraction-free “triangle” primitive.

The Variable Width Δ*d*	0.20 *R*_0_	0.25 *R*_0_	0.30 *R*_0_	0.35 *R*_0_	0.40 *R*_0_
DOE phase	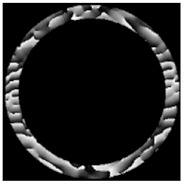	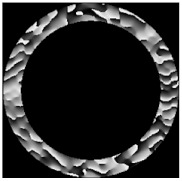	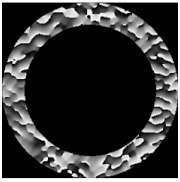	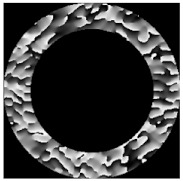	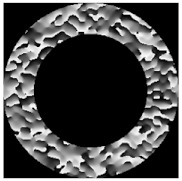
Focal intensity	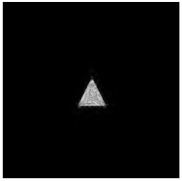	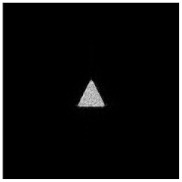	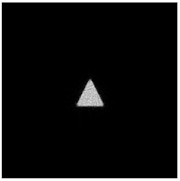	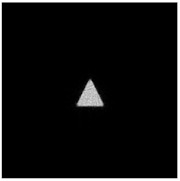	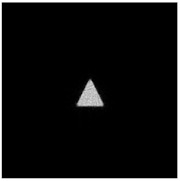
RMSE	0.0019	0.0015	0.0012	0.0009	0.0005

**Table 3:** DOE phase $\psi_{n=90}(x,y)$ with fixed Δ*d =* 0.2 *R*_0_ and variable middle ring radius *d* to form a diffraction-free “triangle” primitive.

The Middle Radius of the Ring *d*	0.5 *R*_0_	0.6 *R*_0_	0.7 *R*_0_	0.8 *R*_0_	0.9 *R*_0_
DOE phase	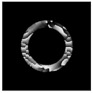	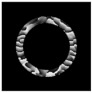	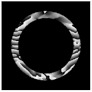	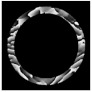	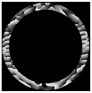
Focal intensity	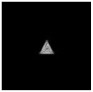	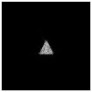	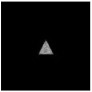	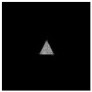	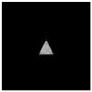
RMSE	0.0028	0.0027	0.0027	0.0026	0.0019

**Table 4:** The comparison of numerical modeling results for a primitive triangle and the composed diffraction-free beam (superposition of simple primitives) defocused from the focal plane (*f* = 100 mm) at different distances Δ*z*.

Type of Beam	Distance to the Focal Plane Δ*z*, mm				
	−75	−50	−25	−15	0
Primitive Triangle with Δ*d* = 0.2 *R*_0_	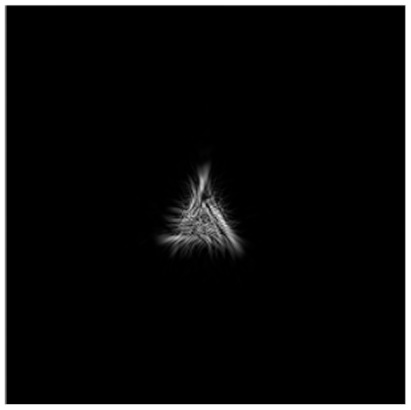	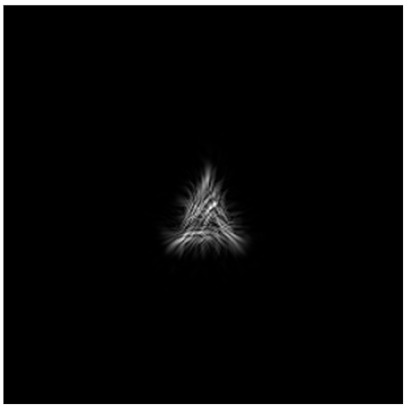	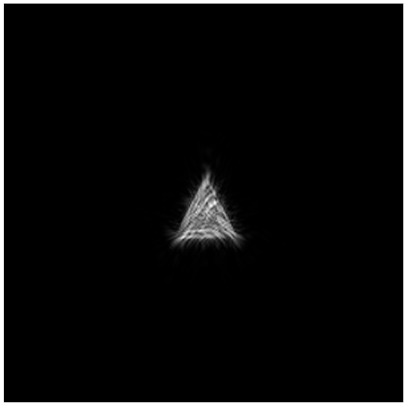	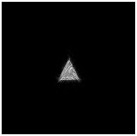	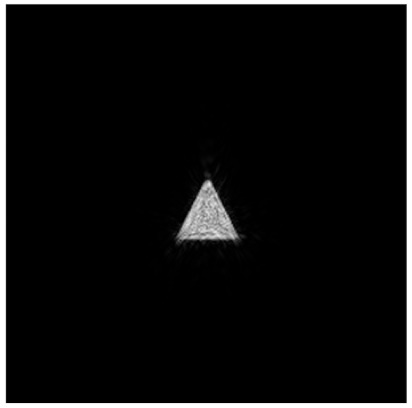
Primitive Triangle with Δ*d* = 0.4 *R*_0_	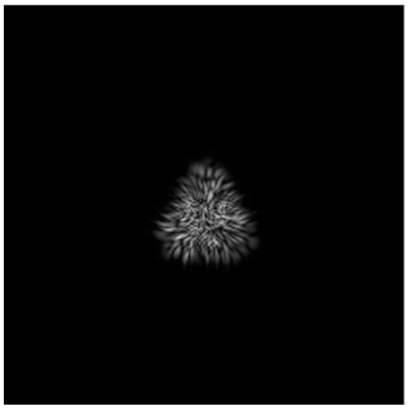	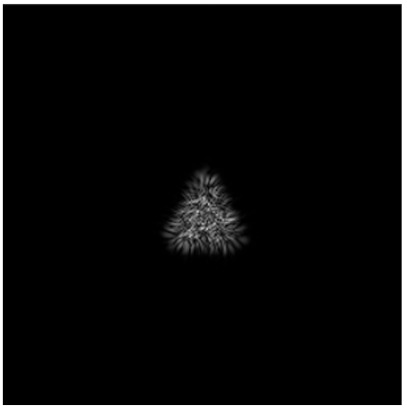	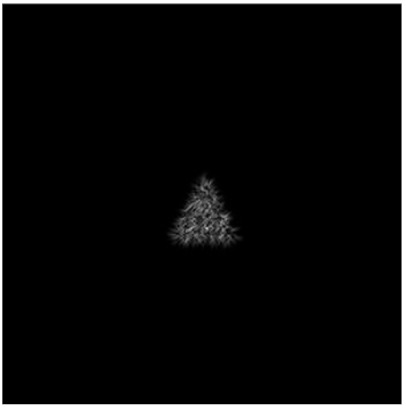	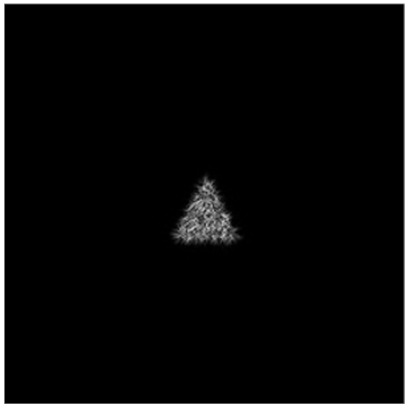	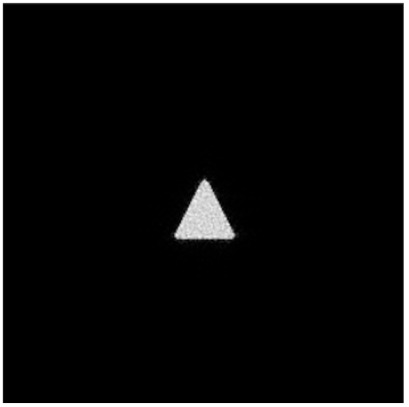
Superposition of simple primitives with Δ*d* = 0.2 *R*_0_	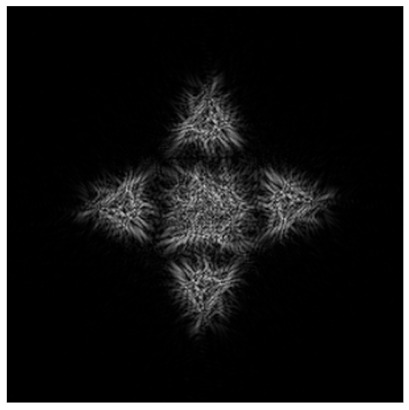	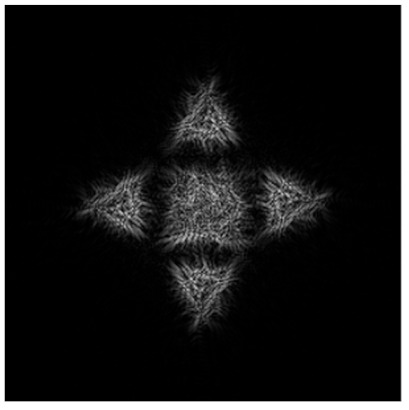	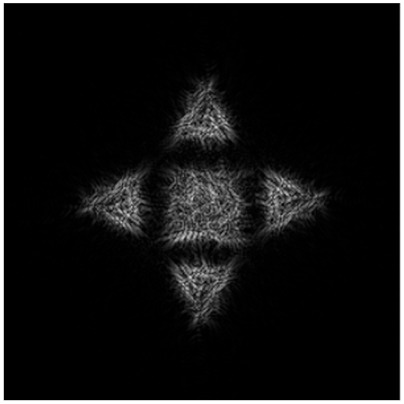	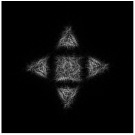	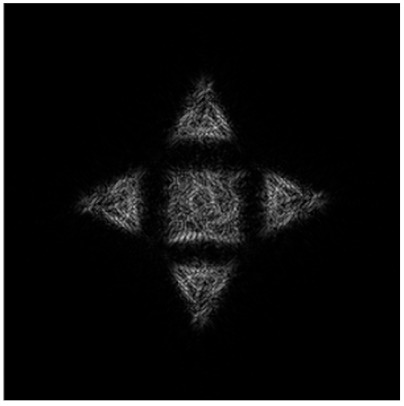

**Table 5:** The comparison of numerical modeling results for a complex pattern *D*(*u,v*) of the “bird” image from the focal plane (*f* = 100 mm) at different distances for Δ*d* = 0.2 *R*_0_ and Δ*d* = 0.4 *R*_0_.

Type of Beam	Distance to the Focal Plane Δ*z*, mm				
	−75	−50	−25	−15	0
Complex pattern *D*(*u,v*) of the “bird” image with Δ*d* = 0.2 *R*_0_	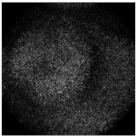	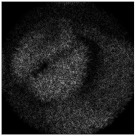	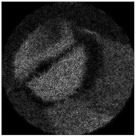	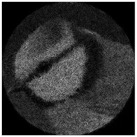	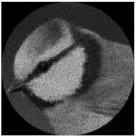
Complex pattern *D*(*u,v*) of the “bird” image with Δ*d* = 0.4 *R*_0_	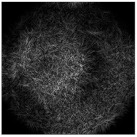	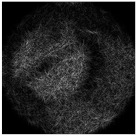	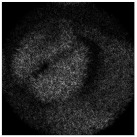	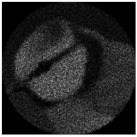	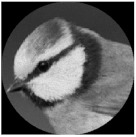

## Data Availability

Data will be made available on request.
